# Eczema is a shared risk factor for anxiety and depression: A meta-analysis and systematic review

**DOI:** 10.1371/journal.pone.0263334

**Published:** 2022-02-18

**Authors:** Qing Long, Hongxia Jin, Xu You, Yilin Liu, Zhaowei Teng, Yatang Chen, Yun Zhu, Yong Zeng

**Affiliations:** 1 Sixth Affiliated Hospital of Kunming Medical University, Yuxi, Yunnan, China; 2 Ziyang Hospital of Traditional Chinese Medicine, Ziyang, Sichuan, China; 3 The First People’s Hospital of Yunnan Province, Affiliated Hospital of Kunming University of Science and Technology, Kunming, Yunnan, China; 4 Second Affiliated Hospital of Kunming Medical University, Kunming, Yunnan, China; Seoul National University College of Medicine, REPUBLIC OF KOREA

## Abstract

Globally, anxiety and depression are the most common psychiatric disorders that add large burdens to individuals and society; however, the mechanisms underlying these disorders are unclear. Several studies have found that eczema is a shared risk factor for both these conditions. We identified and evaluated eligible observational studies from EMBASE and PubMed. In total, 20 relevant cohort and case-control studies comprising 141,910 patients with eczema and 4,736,222 control participants fulfilled our established criteria. Information extracted included study design, location, sample size, sex distribution of cases and controls or reference cohorts, measurements of outcomes, odds ratio (OR) with 95% confidence interval (CI), and adjusted factors for exposure associated with outcome risk. The meta-analysis was performed by calculating the pooled OR with 95% CI, and heterogeneity was assessed using Cochrane Q and I^2^ statistics. The pooled effect showed a positive association (n = 4,896,099, OR = 1.63, 95% CI [1.42−1.88], *p*<0.001) between eczema and depression or anxiety, with positive associations also observed in the depression (n = 4,878,746, OR = 1.64, 95% CI [1.39−1.94], *p*<0.001) and anxiety (n = 4,607,597, OR = 1.68, 95% CI [1.27−2.21], *p*<0.001) groups. Subgroup and sensitivity analyses confirmed that these findings were stable and reliable. This study suggests that eczema is associated with an increased risk of developing depression and anxiety, which may assist clinicians in the prevention or treatment of these disorders.

## Introduction

Anxiety, which is characterized by excessive fear and worry, is a common mental disorder with a high prevalence worldwide. A 2017 global epidemiologic study suggested that approximately 280 million people are diagnosed with an anxiety disorder [1]. Anxiety disorders demonstrate one of the highest non-fatal disease burdens in women, and although awareness is growing rapidly, the prevalence of this disease remains high owing to its misunderstood etiology [1]. Furthermore, an increasing amount of literature proposes that anxiety disorders often coexist with other psychiatric disorders, such as depression and substance abuse [2].

Depression, another common disorder, is characterized by anhedonia, reduced motivation, and disruption of daily activities. Similar to anxiety, the etiology of depression is unclear; thus, its incidence continues to increase. The 1-year prevalence of depression is approximately 6%, while the lifetime prevalence is almost three times higher (15–18%) [3]. Notably, the societal and economic burdens associated with depression are extremely high. Based on a study of global burden of diseases, depressive disorders were characterized as one of the leading causes of years lived with disability for both sexes [1]. Despite both anxiety and depression being harmful to public health, the underlying mechanisms are still unclear. Besides, the etiological and pathological heterogeneity between these two diseases do contribute to the burdens of someone who suffered both of them. Unfortunately, 49~81% depressive patients met criteria of anxiety and 47~88% anxiety individuals can be diagnosed with depression too [4]. Thus, the identification of risk factors shared between anxiety and depression could contribute to the development of effective measures to prevent these diseases.

A growing number of studies have found that eczema is a risk factor for both anxiety and depression [5–7]; however, some researchers have argued that it may not be associated with an increased risk of either disorder [8, 9]. To address this discrepancy, we conducted a meta-analysis that aimed to provide a comprehensive resource for clinicians when making decisions regarding prevention or treatment of these diseases.

## Methods

### Literature search

Two authors (LQ and TZW) formulated the search strategy and separately searched the PubMed and EMBASE databases. The following core search terms were used: “eczema,” “dermatitis, atopic,” “cohort studies,” and “case-control studies.” The detailed search strategy and progress are shown in S1 Method in S1 File. After scanning the literature, the two authors independently extracted the filtered relevant studies. Additionally, the listed references of related meta-analyses and reviews were used as potential resources. The remaining authors were divided into two groups to screen the titles and abstracts of all the extracted articles and to confirm eligibility for further analysis. The following inclusion criteria were used during the full-text review: cohort or case-control studies investigating the relationship between eczema and depression or anxiety, and studies where the exposure was eczema or atopic dermatitis and depression or anxiety was the outcome. Furthermore, the odd ratios (ORs) or risk ratios with 95% confidence intervals (CIs, or sufficient data), were calculated. At least two reviewers independently performed a full-text review of the eligible studies. All disagreements were resolved by the corresponding authors.

### Data extraction

Two authors (LQ and JHX) extracted all data from the included studies after a full-text review. The following items were collected: study identification (defined as first author’s name and the year published), study design, region where the study was conducted, sample size, sex distribution of cases and controls or reference cohorts, measurements of outcomes, ORs with 95% CIs, and adjusted factors for the exposure associated with the risk of outcomes. When a study contained more than one cohort, data for each cohort was extracted separately. In addition, in studies with missing data, we collected or calculated the exact data. The Newcastle-Ottawa Scale (NOS) was used to evaluate the quality of the studies.

### Statistical analyses

This meta-analysis was conducted to identify the association between eczema and depression or anxiety and the combined effect of these two diseases (outcome defined as the presence of any one of the two disorders) by calculating the pooled ORs with 95% CIs. Heterogeneity was assessed by Cochrane Q and I^2^ statistics [10]. If the *p*-value >0.1 and I^2^<50%, the heterogeneity was considered not significant, and a fixed-effects model was applied to calculate the overall effect size (ES); however, when the *p*-value <0.1 or I^2^>50%, the ES was evaluated by a random-effects model. In addition, subgroup analyses were used to explore whether region, study design, sex, conducted years, NOS score, or sample size were contributing factors for heterogeneity. Before conducting the subgroup analysis, we defined small samples as studies with less than 500 participants, medium studies as those with a sample size between 500 and 5,000, and large studies as those with a sample size of more than 5,000 participants. Moreover, high-quality studies were defined as those with a NOS score of 7 or more. We also performed sensitivity analyses to assess the impact of each study on the pooled ES and the stability of the outcome. Furthermore, to judge publication bias, we used Egger’s [11] and Begg’s [12] tests. All modules were performed using STATA software (version 16.0; StataCorp, College Station, TX, USA).

## Results

**Fig 1:**
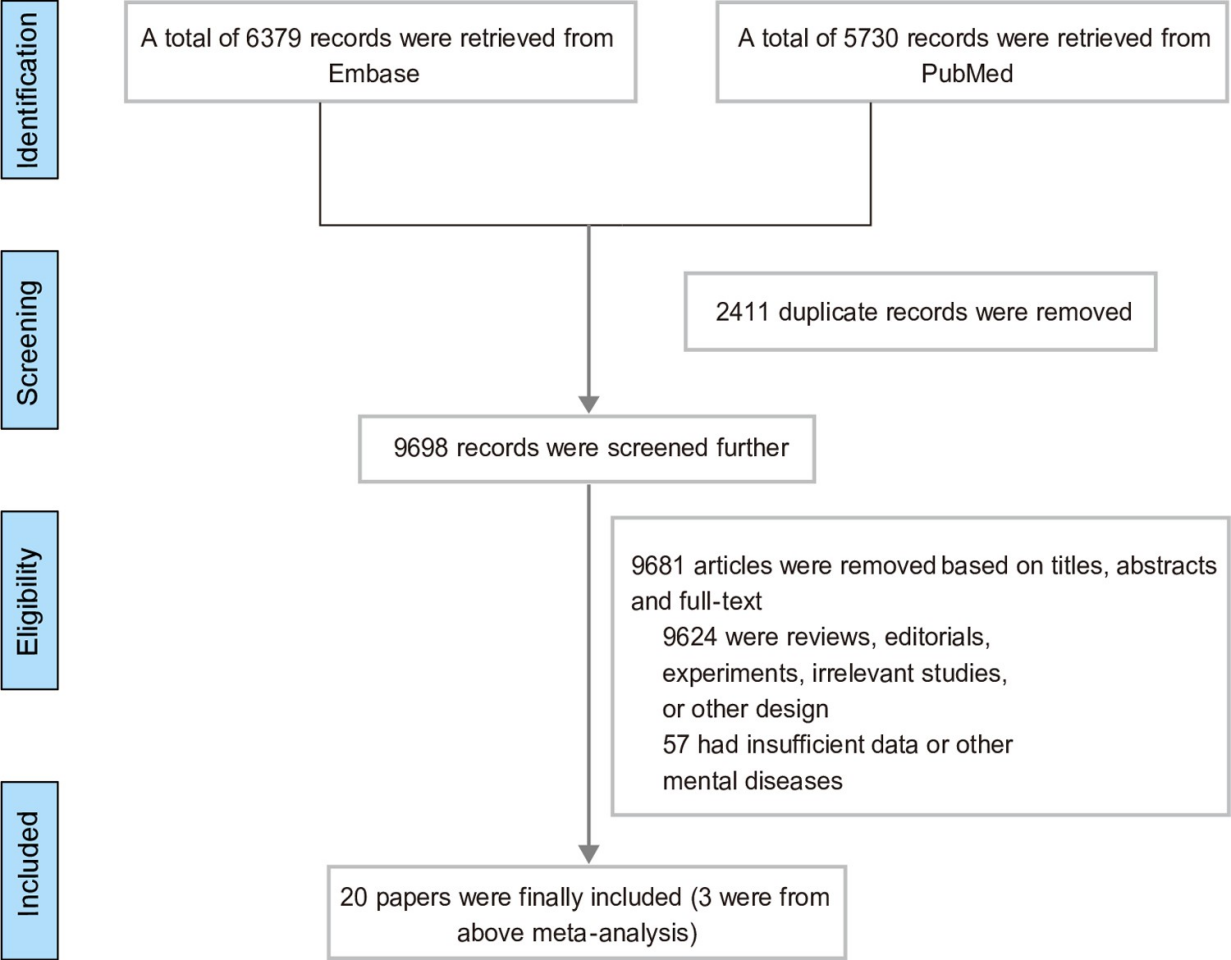
PRISMA flow chart for study selection.

Two databases were searched (PubMed and EMBASE), resulting in identification of 12,109 studies. An additional three studies [13–15] were included from the reference lists of two published meta-analyses [16, 17]. After scanning for duplicates, 2,411 studies were excluded. Then, the titles and abstracts of the remaining 9,698 studies were screened. Thereafter, the full text of 133 studies were reviewed, and 20 eligible studies [9, 13–15, 18–33] were included; the detailed process is shown in Fig 1. Finally, a total of 4,896,099 participants were included. Of these, 18 studies (n = 4,878,746) investigated the association between eczema and the risk of developing depression [9, 13, 15, 18–26, 28–33]. In comparison, nine of the eligible studies (n = 4,607,597) were conducted to explore the association between eczema and anxiety [9, 13, 15, 18, 24, 25, 30, 31, 33]. Detailed information on the characteristics of these studies is shown in Table 1.

**Table 1:** Characteristics of the 20 eligible studies.

Study ID	Study design	Region	Sample size	Age* (years)	Sex (females/males)	OR	95% LCI	95% UCI	Diagnosis of eczema	Diagnosis of Depression/Anxiety	Outcome	Adjustments	Statistical analysis
Shirata, 1996	Case-control	Japan	129	Case: 26.2±7.1	Case: 42/22 Control: 43/22	6.89	3.087	15.38	Patients who were and/or had been treated by a physician during the 1-y period from April 1994 until May 1995	A depressive mood according to the GHQ Slightly affected depressive mood defined as a GHQ score of 3–4; Moderately affected depressive mood defined as a GHQ score ≥5 (Depression subscale)	Depression	NA	Chi-square test
						50.75	6.631	388.388		Defined according to GHQ: Slightly affected defined as a GHQ score of 3–4; Moderately affected defined as a GHQ score ≥ 5 (Anxiety subscale)	Anxiety		
Hashiro, 1997	Case-control	Japan	79	16–57	Case: 26/19 Control: 18/16	1.92	0.747	4.932	Rajka et al. and Yokozeki et al	SDS≥40 (Japanese criteria)	Depression	NA	Chi-square test
						1.533	0.577	4.068			Anxiety		
Zachariae, 2004	Case-control	Denmark	388	Mean age: Case: 30.1 Control: 44.2	Case: 59/36 Control: 187/106	5.862	3.467	9.911	Outpatient clinics of a dermatology department or as hospitalized patients with AD	BDI-II	Depression	NA	Chi-square test
Cvetkovski, 2006	Cohort	Denmark	614	Baseline: 35.8	408/213	0.7	0.3	1.5	Past flexural eczema or currently diagnosed AD by a dermatologist	BDI-II	Depression	Age, sex, diagnostic and subdiagnostic groups, severity, disease duration, socioeconomic status and occupation	Poisson regression models
Schmitt, 2009	Case-control	Germany	7538	≥15	Case: 2644/1125 Control: 2644/1125	1.42	1.13	1.79	ICD-10 L20	ICD-10 F30–39	Depression	Age, sex, presence ⁄ absence of asthma, presence ⁄ absence of allergic rhinitis, and total number of physician visits within study period for reasons other than AE (as an indicator for overall health services utilization)	Multivariate logistic regression
Schmitt, 2011	Cohort	Germany	1529	10	738/791	1.13	0.75	1.72	Physician-diagnosed	German Strength and Difficulties Questionnaire (SDQ), parent version	Depression	Sex, location, household income, breast-feeding, single parent, early pet exposure, day care within infancy, parental history for eczema, pregnancy unplanned/unintended, allergic asthma/allergic rhinitis (ever, physician-diagnosed)	Multivariate logistic regression models
Zachariae, 2012	Case-control	Denmark	40	Case: 31.4±12.7 Control: 41.2±15	Case: 12/8 Control: 16/4	18.379	0.958	352.568	Dermatologists’ diagnosis of AD	BDI-13	Depression	NA	Chi-square test
Covaciu, 2013	Cohort	Sweden	3156 (missing data: 80)	8	1607/1629	2.006	1.454	2.768	Doctors’ diagnosis of eczema from the age of 7	Subscale of EQ-5D	Depression/Anxiety	NA	Chi-square test
Cheng, 2015	Cohort	China	16416	≥12	Case: 3272/4936 Control: 3272/4936	5.097	4.069	6.385	ICD-9-CM: 691 or 691.8	ICD-9-CM codes: 296.2X, 296.3X, 300.4 and 311 ICD-9-CM codes: 300 except 300.04 and 300.03	Depression/Anxiety	NA	Chi-square test
						5.44	3.99	7.44		ICD-9-CM codes: 296.2X, 296.3X, 300.4 and 311	Depression	Adjusted by demographic data and allergic comorbidities and atopic dermatitis as a binary variable	Cox regression model
						3.57	2.55	4.98		ICD-9-CM codes: 300 except 300.04 and 300.03	Anxiety		
Catal, 2016	Case-control	Turkey	154	3–5	Case: 42/38 Control: 33/41	0.586	0.198	1.734	Hanifin and Rajka criteria	ECI-4	Depression/Anxiety	NA	Chi-square test
						0.456	0.04	5.133			Depression		
						0.638	0.193	2.106			Anxiety		
Drucker, 2016	Case-control	USA	65612	Mean age: 48	Case: 10006/6397 Control: 30017/19192	1.541	1.445	1.644	According to ICD-9 code 691.8	NA	Depression	NA	Chi-square test
						1.683	1.572	1.803			Anxiety		
Nanda, 2016	Cohort	USA	546	7	247/299	0.6	0.2	1.9	≥1 aeroallergen SPT positive and frequent skin scratching for 6 months and 1 other symptom for 6 months (redness/red spots, raised bumps, or rough dry skin) at age 4 years	Depression subscale BASC-2 T score >59	Depression	NA	Logistic regression
						1.4	0.7	3.1		Anxiety subscale BASC-2 T score >59	Anxiety		
Johansson, 2017	Cohort	Sweden	3606	10–18	Females and males	0.84	0.63	1.12	Parental questionnaires and include report of dry skin in combination with itchy skin lesions on age-specific locations and/or reported doctor’s diagnosis of eczema in the latest 12 months	Based on prescribed drugs for depression derived from Swedish Prescribed Drug Register (SPDR)	Depression	NA	Logistic regression
Brew, 2018	Cohort	Sweden	14197	9	7036/7161	1.23	1.09	1.38	Parental questionnaire	Screen for Child Anxiety Related Emotional Disorders (SCARED) and Shortened Mood and Feelings (SMFQ) questionnaires answered by parents	Depression/Anxiety	Sex, gestational age, birth weight, maternal age at delivery, parent’s birth country	NA
Choi, 2018	Case-control	Korea	42222	≥19	Case: 12495/8616 Control: 12477/8634	2.36	1.48	3.74	Self-reported AD diagnosis	Self-reported diagnosis of depression by a doctor at any point in the respondent’s lifetime	Depression	Age, sex, current smoking status, current drinking status, preference for low sodium intake, BMI, and year	Multivariable regression model
Sato, 2018	Cohort	Sweden	201090	17–20	Males only	1.43	1.21	1.69	Based on the record review and diagnoses conducted by a physician at the conscription assessment	Depression defined as the sum of dispensed antidepressant equivalent to a use for 180 d within 365 d in the Prescription Register	Depression	NA	Cox proportional hazards regression
Thyssen, 2018	Cohort	Denmark	4269495	Case: 38.3±13(mild) 42.8±15(moderate to Severe) Reference: 48±17.9 Case: 38.3±13(mild)	Case: 6465/3573 Reference: 2171073/2088384	1.531	1.231	1.903	According to either ICD-8 code 691 or ICD-10 code L20 given by a dermatologist	Depression defined according to either ICD-8 code 296.09, 296.29, 296.99, 298.09, 300.19, or 300,49 or ICD-10 code F32-33 Anxiety defined according to either ICD-8 code 300.0, 300.2, or ICD-10 code F40-41	Depression/Anxiety	NA	Chi-square test
						1.479	1.134	1.928		According to either ICD-8 code 296.09, 296.29, 296.99, 298.09, 300.19, or 300,49 or ICD-10 code F32-33	Depression		
						1.643	1.125	2.398		According to either ICD-8 code 300.0,300.2, or ICD-10 code F40-41	Anxiety		
Kauppi, 2019	Case-control	Finland	98053	Case: 31.8±16.9 Control: 45.7±19.4	Case: 36460/21230 Control: 25832/14531	1.398	1.346	1.452	ICD-9 code 6918B and ICD-10 code L20.0	Depression according to Finnish version of ICD-9 2961, 2968A, 3004A; ICD-10 F32, F33, F34.1 Anxiety according to Finnish version of ICD-9 3000A–C, 3002B–D, 3002X, 3003A; ICD-10 F40–F42	Depression/Anxiety	NA	Chi-square test
						1.23	1.17	1.29		According to Finnish version of ICD-9 2961, 2968A, 3004A; ICD-10 F32, F33, F34.1	Depression	Age and sex	
						1.14	1.07	1.22		According to Finnish version of ICD-9 3000A–C, 3002B–D, 3002X, 3003A; ICD-10 F40–F42	Anxiety		
Teichgräber, 2021	Case-control	Germany	14122	5–17	Case: 3771/3290 Control: 3771/3290	1.50	1.37	1.64	ICD-10. L20-L30	ICD-10: F32, F33, F41.2	Depression	NA (cases were matched to controls) P-values was corrected using the Bonferroni adjustment method	Multivariable regression mode
					Females only	1.43	1.26	1.61					
					Males only	1.58	1.39	1.80					
Vittrup, 2021	Cohort	Denmark	157113	0–17	Case: 8138/6145 Reference: 81380/61450	0.50 1.29	0.18 0.84	1.42 2.00	ICD-10 code L20	ICD-10 code F32-33 ICD-10 code F40-41	Depression Anxiety	sex, age, socioeconomic status, country of origin, somatic comorbidities, and the variable asthma/hay fever/food allergy	Cox regression models

NA: Not available in original studies.

*Age: The single digits refer to the subjects of original studies with a specific age.

### Eczema and depression or anxiety

**Fig 2:**
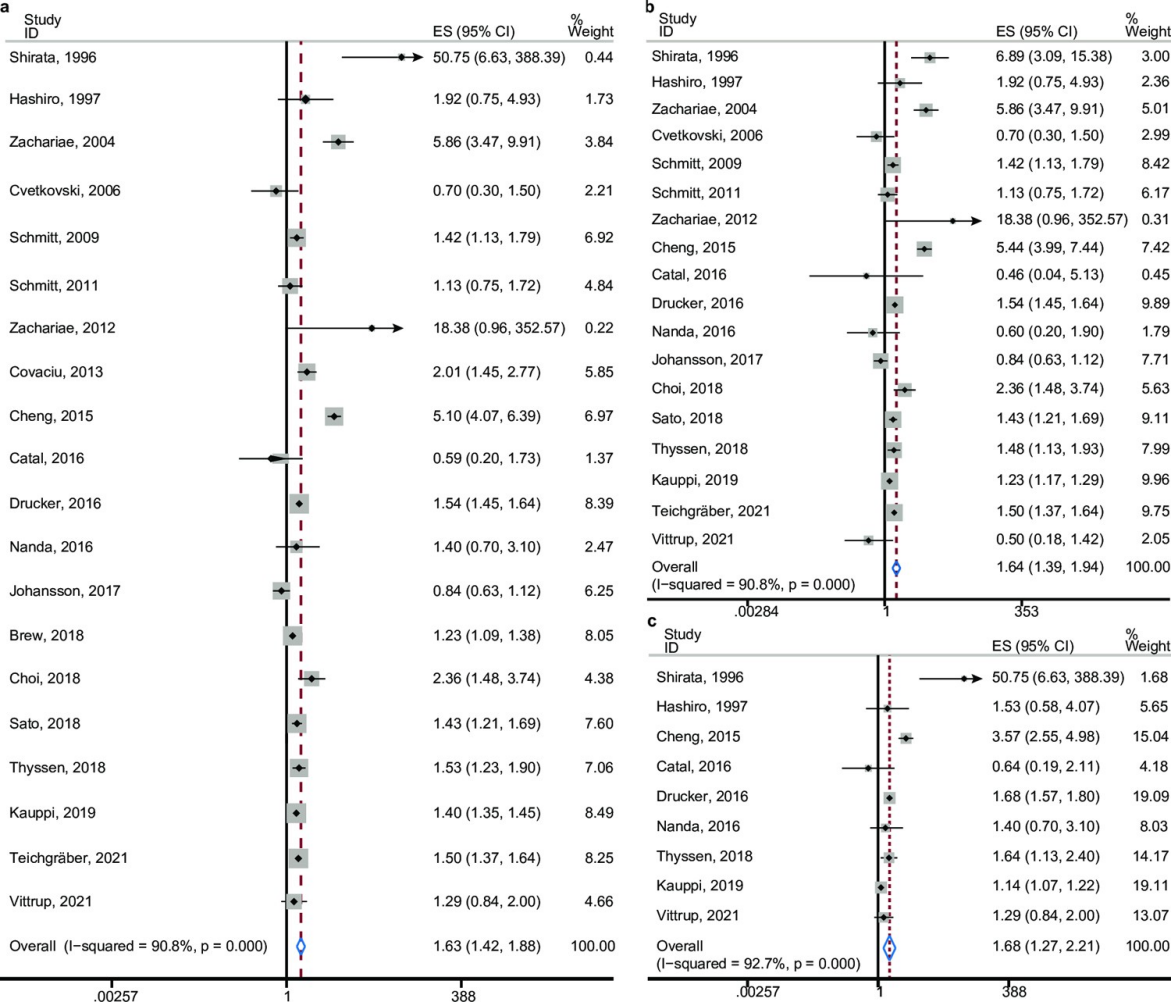
Forest plots of the three groups. Forest plot of eczema associated with (**a**) anxiety/depression, (**b**) depression, and (**c**) anxiety groups. The pooled effect size and 95% confidence interval are indicated with a white diamond. The effect size and 95% confidence interval for each study are indicated by a black diamond and black line. The gray squares refer to the weight of each study calculated in the pooled effect size.

A total of 20 studies were included in this analysis. Of these, 10 were prospective cohort studies [9, 14, 20, 22, 24, 26, 27, 29, 30, 33] and another 10 were case-control studies [13, 15, 18, 19, 21, 23, 25, 28, 31, 32]. Furthermore, the originating regions included Asia (n = 5 [13, 18, 24, 25, 28], Europe (n = 13 [14, 19–23, 26, 27, 29–33], and North America (n = 2 [9, 15]. The pooled ES indicated that there was a positive association between eczema and the risk of developing depression/anxiety (OR = 1.63, 95% CI [1.42, 1.88], *p*_(ES)_<0.001); however, significant heterogeneity was also observed (*p*<0001, I^2^ = 90.8%; Fig 2A).

**Fig 3:**
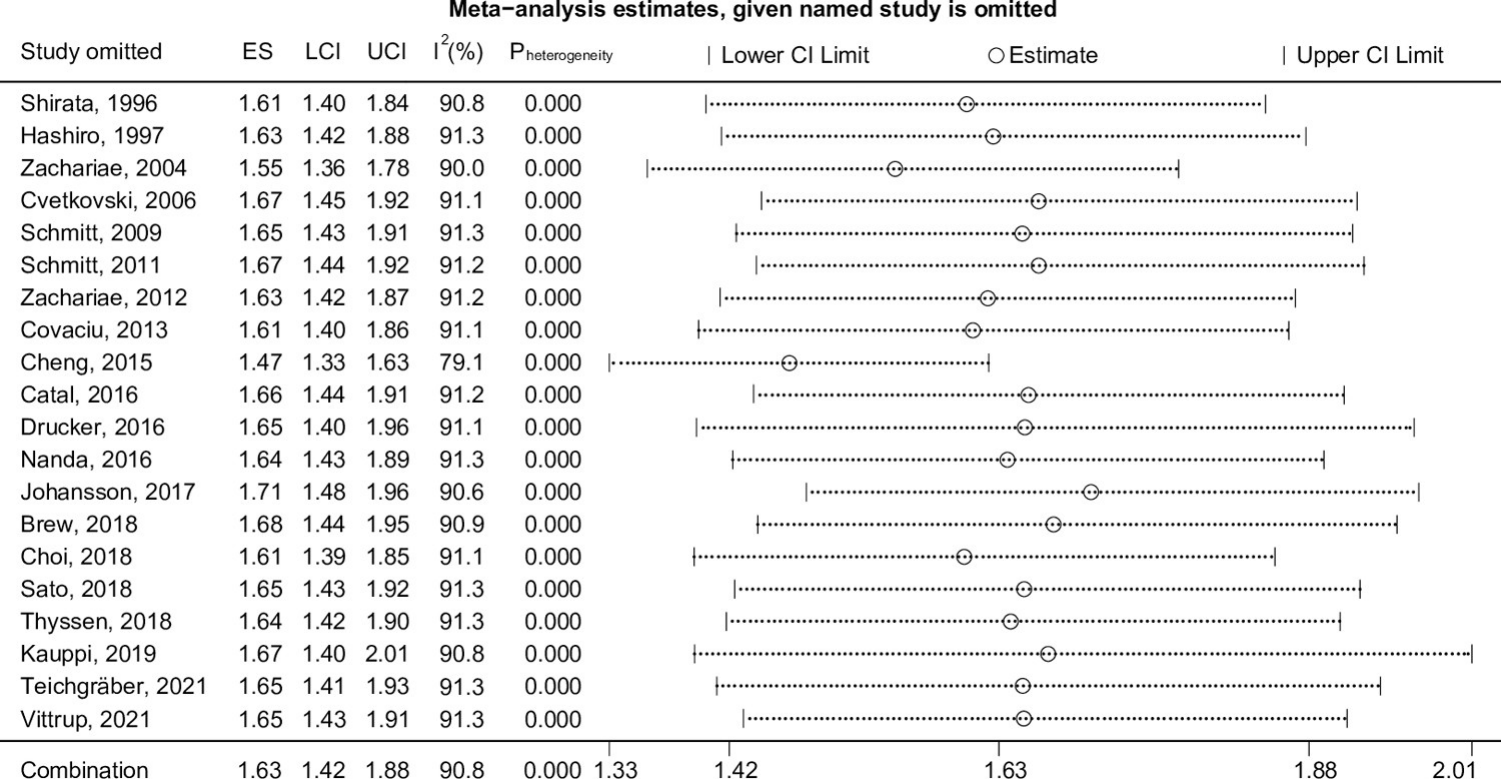
Sensitivity analysis of eczema associated with anxiety/depression.

To identify the potential influencing factors that contributed to the heterogeneity, we performed a subgroup analysis. In consideration of the different impact of eczema on depression/anxiety between males and females, the gender was chosen to perform subgroup analysis. While the significant difference was not shown between males and females. Additionally, it was found that the studies without analyzing males and females separately mainly contributed the heterogeneity (*p* < 0001, I^2^ = 94.0%, eFig 1a in S1 File). However, there were only one study analyzed the effect on females and only two studies analyzed males. We found that studies from North America showed no contribution to heterogeneity (I^2^ = 0.0%, *p* = 0.801), and low-quality studies also showed a smaller trend of heterogeneity; however, the *p*-value and I^2^ were still significant (I^2^ = 57.1%, *p* = 0.022; eFig 1a in S1 File). Nevertheless, after removing the studies with a sample size less than 500 [13, 18, 19, 23, 25], the subgroup analysis showed that cohort studies may be the contributing factor for heterogeneity (*p*<0001, I^2^ = 94.0%; eFig 1b in S1 File). In addition, the association remained positive (pooled OR = 1.54, 95% CI [1.35, 1.75], *p*_(ES)_<0.001). As all the deleted studies were case-controlled, a sensitivity analysis was conducted, revealing that the ES did not change considerably, regardless of whether the studies were excluded (Fig 3).

### Eczema and depression

Eighteen studies were analyzed to assess the interaction between eczema and depression. Of these, eight were cohort studies [9, 20, 22, 24, 26, 29, 30, 33] and 10 were case-control studies [13, 15, 18, 19, 21, 23, 25, 28, 31, 32]. More than 141,910 patients with eczema and 4,736,222 control participants were included in our meta-analysis. In addition, five studies [13, 18, 24, 25, 28] originated from Asia, 11 [19–23, 26, 29–33] originated from Europe, and two [9, 15] originated from North America. Our results indicated that the association between eczema and the risk of developing depression was significantly positive (OR = 1.64, 95% CI [1.39, 1.94], *p*_(ES)_<0.001). Substantial heterogeneity was also observed for this comparison (*p*<0.001, I^2^ = 90.8%; Fig 2B).

In the subgroup analysis, compared with other regions and high-quality studies, contributions from North America (I^2^ = 62.8%, *p* = 0.101) or studies with low-quality (I^2^ = 56.6%, *p* = 0.032) appeared relatively smaller; however, statistically, this was difficult to interpret. When the study design was chosen as the subgroup, the heterogeneity was attributable to small (*p* = 0.053, I^2^ = 57.3%) and large sample size (*p*<0.001, I^2^ = 93.3%) studies (detailed data shown in eFig 1 in S1 File). The heterogeneity of studies with a medium sample size was not statistically significant (*p* = 0.524, I^2^ = 0%). The conducted year of these studies can’t distinguish the period that contributed to the heterogeneity mainly. Furthermore, after study removal, the ES did not fluctuate in the sensitivity analysis (eFig 2a in S1 File). When the different impact of eczema on depression/anxiety between males and females was taken into consideration, the results showed that the studies without separating females and males into two groups were a major contributing factor to heterogeneity (eFig 1a in S1 File). And significant difference between males and females was not found.

### Eczema and anxiety

Nine studies included in the analysis examined the relationship between eczema and anxiety. They comprised five case-control studies [13, 15, 18, 25, 31] and four cohort studies [9, 24, 30, 33]. A total of 106,894 patients with eczema and 4,500,703 control participants were included. In addition, four studies [13, 18, 24, 25] were from Asia, three [30, 31, 33] were from Europe, and another two [9, 15] were from North America. Our meta-analysis showed that eczema was significantly associated with an increased risk of developing anxiety (pooled OR = 1.68, 95% CI [1.27, 2.21], *p*_(ES)_<0.001). Similar to the other two meta-analyses, significant heterogeneity was observed (*p*<0.001, I^2^ = 92.7%; Fig 2C).

When the NOS score was selected for the analysis, we found that low-quality studies had a very limited contribution to the heterogeneity (*p* = 0.479, I^2^ = 0.0%); however, the weight of these studies (17.85%) was far less than that of those of relatively high quality (82.15%). When region was considered, the subgroup analysis results showed that studies from Europe (*p* = 0.155, I^2^ = 46.4%) and North America (*p* = 0.629, I^2^ = 0.0%) may have limited contributions to the heterogeneity. Furthermore, the sensitivity analysis revealed that, regardless of the studies, the ES did not change significantly (eFig 2b in S1 File).

### Publication bias

**Fig 4:**
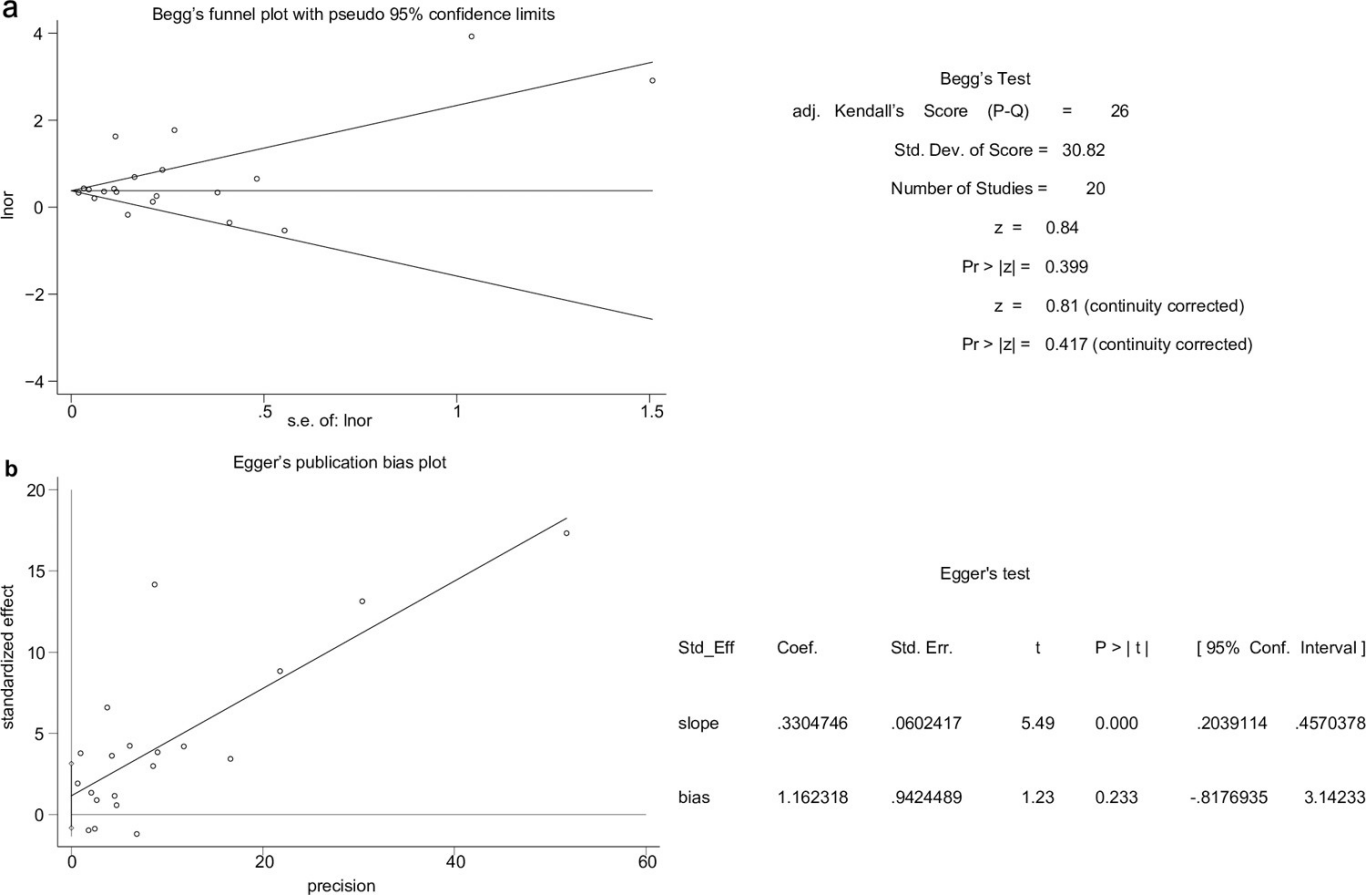
Publication bias assessment of eczema associated with anxiety/depression. (**a**) The horizontal line refers to pooled effect estimates, and the two oblique lines indicate the pseudo 95% confidence intervals. (**b**) Egger’s test showed the absence of significant publication bias concerning eczema associated with anxiety/depression. Detailed information for both the tests is shown on the right side.

For the eczema and depression/anxiety groups, the Begg’s (*p* = 0.417) and Egger’s tests (*p* = 0.233) revealed no significant bias (Fig 4). Moreover, evidence of publication bias in the eczema and depression groups was not observed (Begg’, *p* = 0.88; Egger, *p* = 0.242). These analyses were not performed in the eczema and anxiety groups owing to the limited number of studies included.

## Discussion

Depression and anxiety are the most common psychiatric disorders worldwide, and both result in heavy burdens to society and the afflicted individuals. Several studies have found that eczema is associated with an increased risk of developing depression or anxiety [16, 17, 34]; however, many cross-sectional studies were included in these analyses. Therefore, it is difficult to clarify the impact of an eczema diagnosis. In addition, they failed to consider the combined effect of depression and anxiety. Thus, we conducted this meta-analysis with only case-control and cohort studies to verify the potential causal relationship between eczema and depression/anxiety.

The main finding of this meta-analysis was that there was a significant and positive association between eczema and the risk of depression or anxiety. A positive association was also found between eczema and depression/anxiety. Despite the differing inclusion criteria, the pooled ES was consistent with two similar meta-analyses [16, 34]. These results indicate that eczema is a shared risk factor for depression and anxiety. Ronnstad et al. hypothesized that burdens caused by eczema (e.g., itching, disrupted sleep, and social isolation) contributed to an increased risk of developing depression or anxiety [16]; however, the potential mechanism underlying these effects is still unclear.

Several studies have demonstrated that depression and anxiety are often concomitant [35–37]. A population-based study conducted by Shafiee et al. indicated that the symptoms of depression and anxiety may be associated with excessive oxidative stress [38]. In addition, their previous cohort study revealed an association between enhanced inflammatory states and increased depression and anxiety scores based on Beck Depression and Anxiety Inventories [39]. Furthermore, Vogelzangs found that the expression of cytokines in response to *ex vivo* stimulation of blood by lipopolysaccharides is positively associated with current or previous depression/anxiety, and some therapies can improve the symptoms of both disorders [40]. Therefore, researchers have suggested that the two disorders share some common risk factors [41]. Moreover, studies have found that oxidative stress is increased and antioxidant capability is decreased during the exacerbation of eczema [42]. Additionally, as an inflammatory skin disease, eczema is characterized by immune dysregulation and inflammatory activation [43], and the expression of many proinflammatory factors is upregulated in the peripheral blood of patients with eczema [44]. Therefore, these data suggest that eczema is associated with depression and anxiety at the physiological level. In combination with the results of this study, these data suggest that eczema may increase the risk of depression and anxiety through these common pathways.

Our results also show that eczema may be a risk factor for depression, which is consistent with recent studies, although the designs of these studies differed [45–47]; however, the relationship between eczema and depression in our present findings differ from those of Slattery et al. who conducted a community-based analysis [8]. The possible reasons for this discrepancy include the following: first, the sample size of the study was significantly lower than that of most of the studies included in our meta-analysis; second, their participants were from a specific community, while the participant selection criteria varied greatly among our included studies; and finally, the diagnosis of eczema and depression differed significantly. Moreover, another population-based study with a larger sample size supports our finding [48]. Based on the growing body of evidence, we conclude that eczema has a negative role in the development of depression.

A positive association between eczema and anxiety was found in a recent cohort study with many participants [7]. In addition, a European dermatological multicenter study demonstrated that patients with hand eczema had higher anxiety scores [49]. This meta-analysis was in accordance with those studies and indicated that eczema was related to the development of anxiety. Although the mechanism is not completely understood, the following studies offer some valuable insight. First, an animal experiment conducted by Yeom et al. found that mice with atopic dermatitis-like skin lesions also displayed anxiety-like and depression-like behaviors, which were associated with neuroplasticity-related changes in reward circuitry [50]. Second, Li et al. found that atopic dermatitis-like and anxiety-like symptoms in BALB/c mice can be reduced by the antidepressant fluoxetine [51]. Finally, treating patients with eczema with dupilumab can ameliorate the skin condition and improve symptoms of anxiety [52]. Thus, it can be inferred that there may be some shared mechanisms between eczema and anxiety. Therefore, eczema may be a risk factor for the pathogenesis of anxiety.

Considering the high heterogeneity, a subgroup analysis was performed among the three groups. First, the subgroup analysis showed that the heterogeneity was mainly from cohort studies after excluding several case-control studies with small sample sizes in the depression/anxiety group. This suggests that sample size was the principal contributor to the heterogeneity demonstrated between the case-control studies. Moreover, owing to a variety of influencing factors that may result in differences, heterogeneity appeared more obvious between the cohort studies. Second, in the depression group, the heterogeneity arising from the small and large sample sizes can be explained in a similar way to that of the depression/anxiety group. The large sample size studies mainly comprised cohort studies. Lastly, in the anxiety group, relatively high-quality studies, owing to the various sample sizes and different study designs, contributed most to the heterogeneity; however, the sensitivity analysis of the three groups also showed that our results were stable.

There are several strengths in this meta-analysis. First, most of the included studies were cohort-based, which has been considered the most conceivable observational study design when identifying causal relationships or risk factors [53]. Second, the stable results of the sensitivity analysis confirmed that our findings were reliable. Finally, there was no significant publication bias demonstrated. Our meta-analysis is not without limitations. First, obvious heterogeneity was observed in the three groups; however, after performing subgroup analysis, potential contributors were identified in each group. In addition, the sensitivity analysis verified the reliability of our results. Second, the association between the severity of eczema and depression or anxiety could not be clarified due to limited information. Third, the data could not be completely adjusted for probable confounders. Finally, the mechanisms underlying our findings are still not fully understood.

In summary, our results demonstrated that eczema is a risk factor for developing depression or anxiety. Given the growing number of patients with both these disorders, our findings are significant and warrant adoption of relevant protective/preventative measures; however, as the mechanisms are still unclear, further well-designed studies are needed to reveal the potential molecular signaling pathways underlying the present results.

## Supporting information

S1 Data (XLSX)

S1 File (DOCX)
