# Supplementary material for: Eczema is a shared risk factor for anxiety and depression: A meta-analysis and systematic review
Source: PLoS One. 2022 Feb 18;17(2):e0263334. doi: 10.1371/journal.pone.0263334 (PMC8856547; doi:10.1371/journal.pone.0263334)
Supplement: S1 File — (DOCX) [file pone.0263334.s002.docx]

**Supplementary Materials**

**S1.** Details concerning the search strategies and process

**Database: PubMed (from inception to March 31, 2021)**

Search strategy:

Terms specific to eczema

#1. "Eczema"[Mesh] OR "Eczema, Dyshidrotic"[Mesh] OR "Dermatitis, Atopic"[Mesh]

#2. (((((((((((((((((((((((((((((((((Eczemas[Text Word]) OR (Dermatitis, Eczematous[Text Word])) OR (Dermatitides, Eczematous[Text Word])) OR (Eczematous Dermatitides[Text Word])) OR (Eczematous Dermatitis[Text Word])) OR (Dyshidrotic Eczema[Text Word])) OR (Dyshidrotic Eczemas[Text Word])) OR (Eczemas, Dyshidrotic[Text Word])) OR (Vesicular Palmoplantar Eczema[Text Word])) OR (Eczema, Vesicular Palmoplantar[Text Word])) OR (Eczemas, Vesicular Palmoplantar[Text Word])) OR (Palmoplantar Eczema, Vesicular[Text Word])) OR (Palmoplantar Eczemas, Vesicular[Text Word])) OR (Vesicular Palmoplantar Eczemas[Text Word])) OR (Pompholyx[Text Word])) OR (Eczema, Dyshydrotic[Text Word])) OR (Dyshydrotic Eczema[Text Word])) OR (Dyshydrotic Eczemas[Text Word])) OR (Eczemas, Dyshydrotic[Text Word])) OR (Atopic Dermatitides[Text Word])) OR (Atopic Dermatitis[Text Word])) OR (Dermatitides, Atopic[Text Word])) OR (Neurodermatitis, Atopic[Text Word])) OR (Atopic Neurodermatitides[Text Word])) OR (Atopic Neurodermatitis[Text Word])) OR (Neurodermatitides, Atopic[Text Word])) OR (Neurodermatitis, Disseminated[Text Word])) OR (Disseminated Neurodermatitides[Text Word])) OR (Disseminated Neurodermatitis[Text Word])) OR (Neurodermatitides, Disseminated[Text Word])) OR (Eczema, Atopic[Text Word])) OR (Atopic Eczema[Text Word])) OR (Eczema, Infantile[Text Word])) OR (Infantile Eczema[Text Word])

#3. #1 OR #2

Terms specific to study design

#4. ("Cohort Studies"[Mesh]) OR ("Case-Control Studies"[Mesh]) OR ("risk"[Mesh]) OR ((hazard [Text Word] OR odds [Text Word]) AND ratio*[Text Word]) OR (relative [Text Word] AND risk [Text Word])

Combination of terms to identify eczema

#5. #3 AND #4

#6. #5 AND ("case reports"[pt] OR letter[pt] OR review[pt] OR editorial[pt] OR comment[pt] OR "practice guideline"[pt] OR "historical article"[pt] OR news[pt] OR meta-analysis[pt])

#7. #5 NOT #6

**Database: Embase (from inception to March 31, 2021)**

Search strategy:

Terms specific to eczema

#1. 'eczema'/exp OR 'eczema’: ti,ab

#2. 'atopic eczema'/exp OR 'atopic eczema’: ti,ab

#3. 'dyshidrotic eczema'/exp OR 'dyshidrotic eczema’: ti,ab

#4. 'atopic dermatitis'/exp OR 'atopic dermatitis’: ti,ab

#5. #1 OR #2 OR #3 OR #4

Terms specific to study design

#6. 'cohort study'/exp OR (cohort stud*): ti,ab

#7. 'case-control study'/exp OR 'case-control stud*':ti,ab

#8. 'risk'/exp OR risk: ti,ab OR 'ratio'/exp OR ratio:ti,ab

#9. #6 OR #7 OR #8

Combination of terms to identify eczema

#10. #5 AND #9

#11. #10 AND ([article]/lim OR [article in press]/lim) AND [humans]/lim AND [clinical study]/lim AND [embase]/lim

PubMed:

Studies count: 5730

Embase:

Studies count: 6379

**S2.** Figures

**eFigure 1. Subgroup Analysis**

**
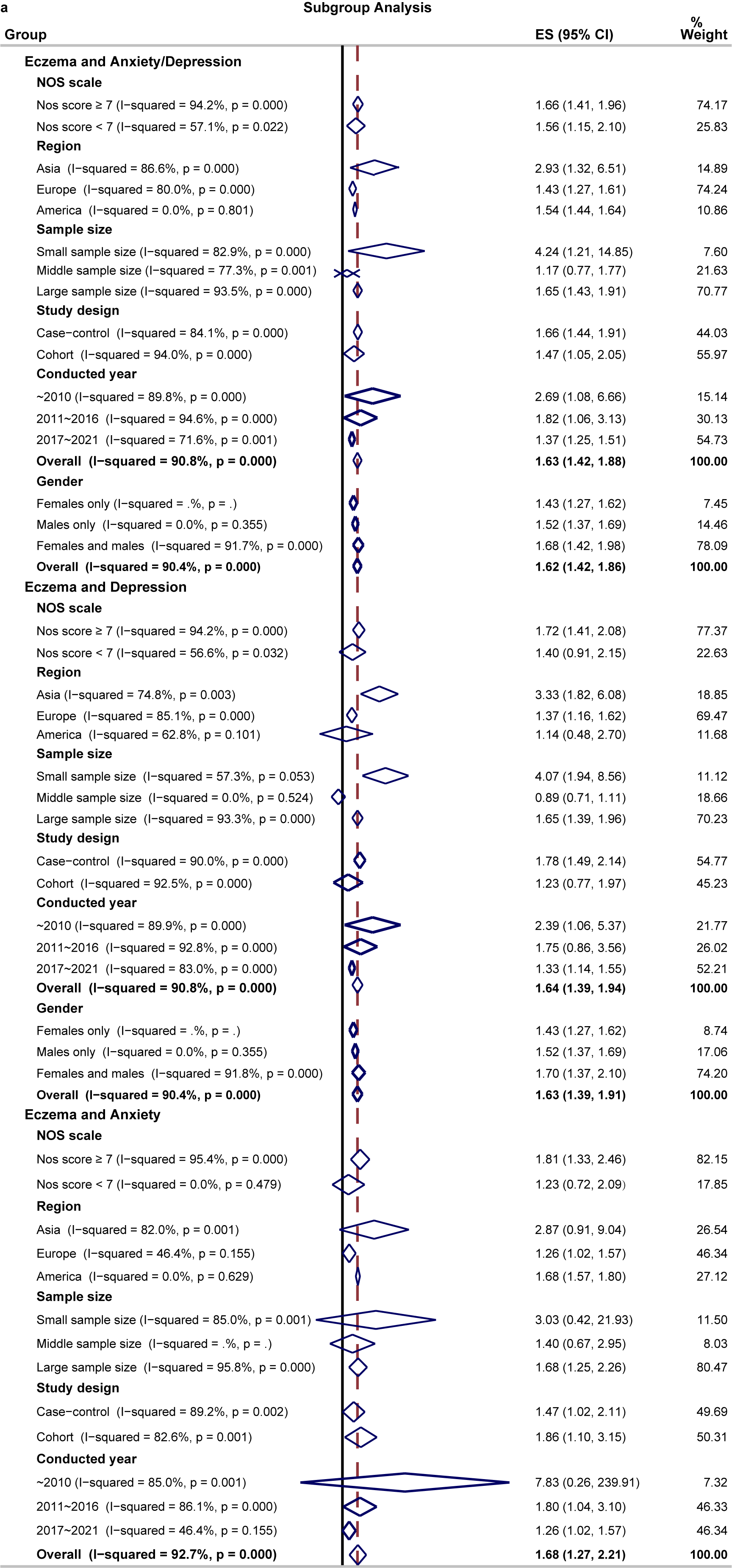
**


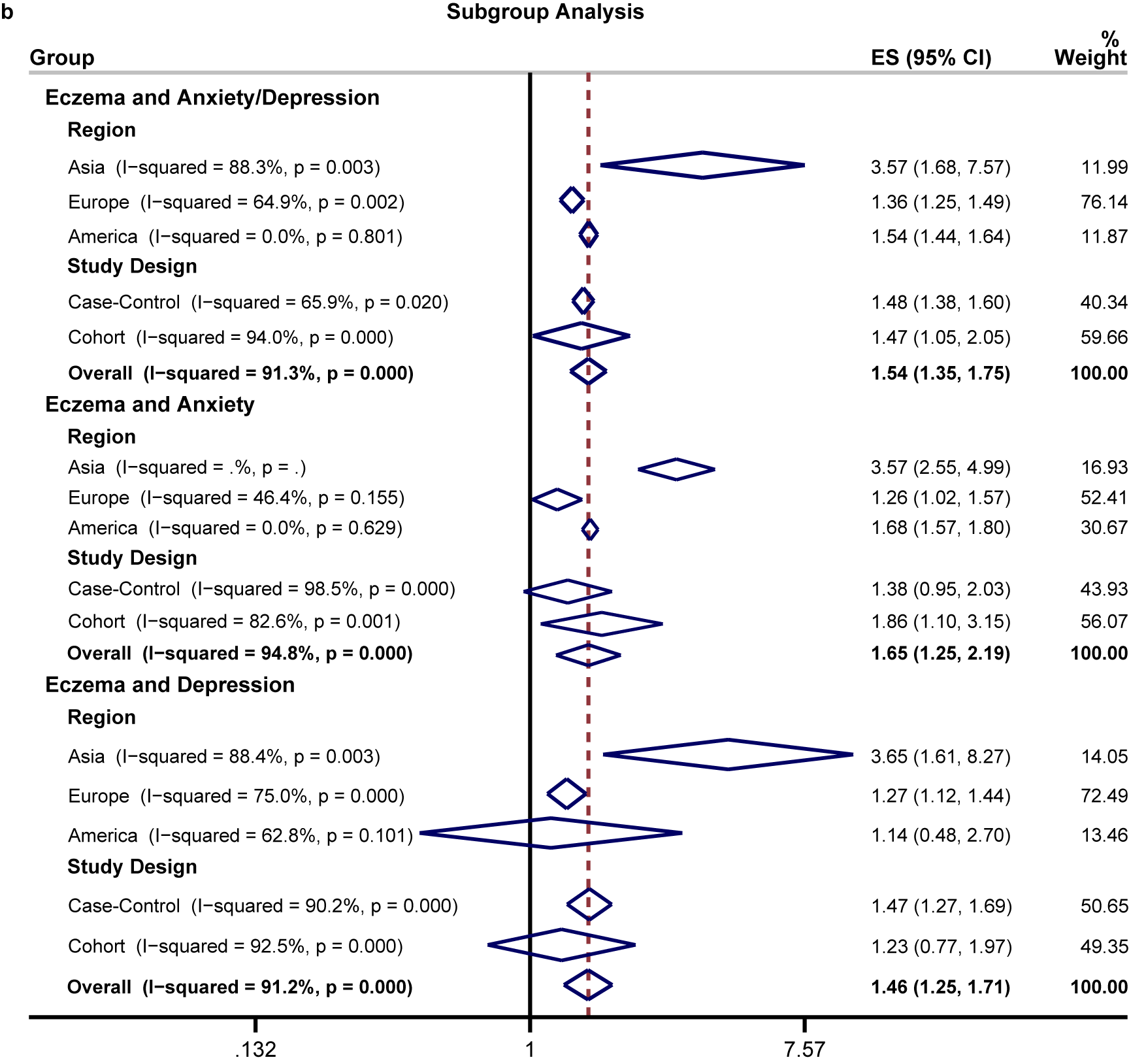


**Note:** Weights are from random effects analysis.

**a.** Subgroup analysis of original studies. **b.** Subgroup analysis after deleting sample size less than 500. The groups referred to the association between eczema and anxiety/depression, depression, anxiety and eczema separately. The bold subtitle referred to different subgroup which was chosen to be analyzed. The gender subgroup analysis included one study with males and females cohort separately. Thus, the overall pooled effects are little different with previous results.

**eFigure 2. Sensitivity analysis**


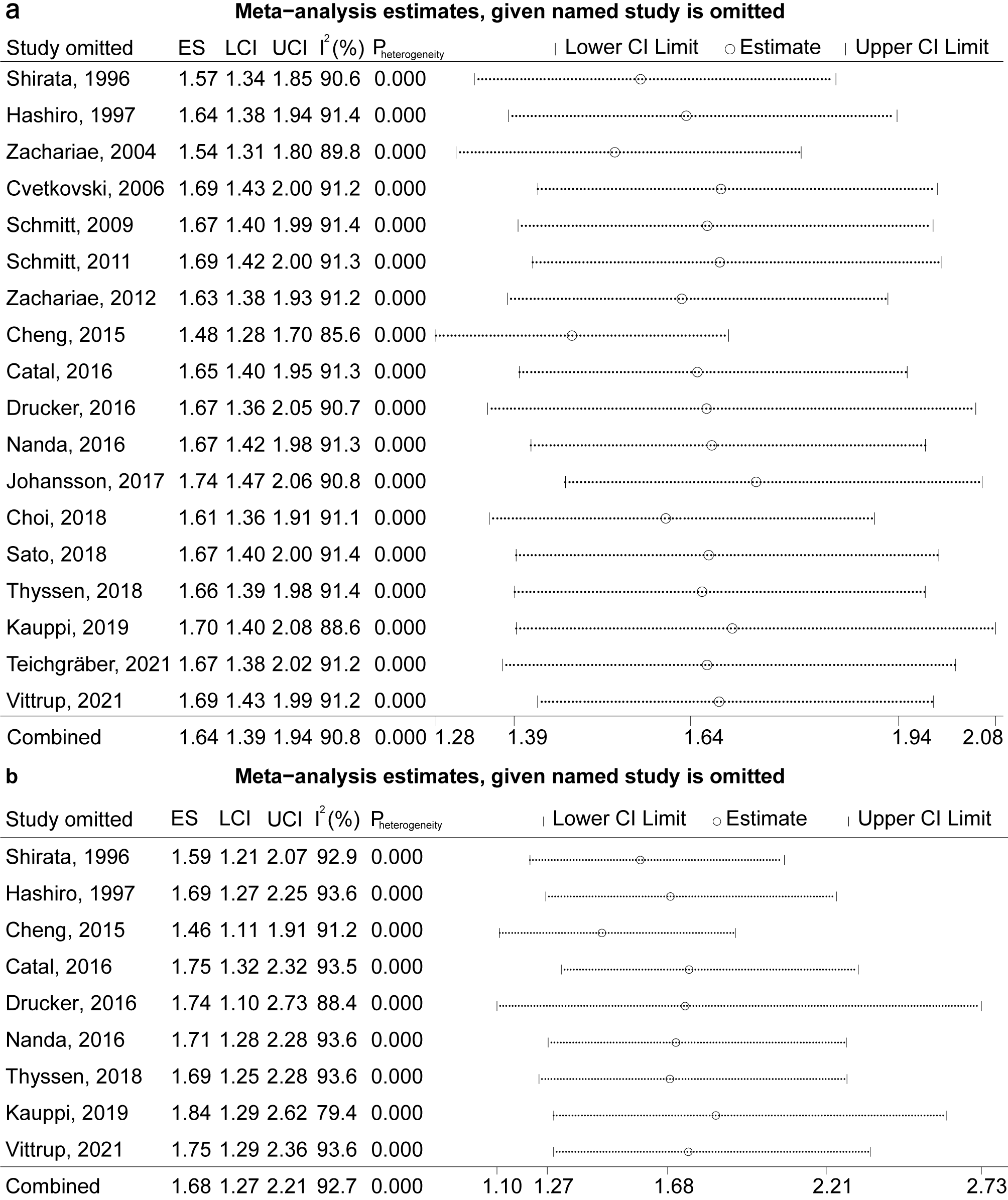


**a**. Sensitivity analysis of the association between eczema and depression. **b**. Sensitivity analysis of the association between eczema and anxiety.

**eFigure 3. Publication bias assessment of eczema associated with depression**


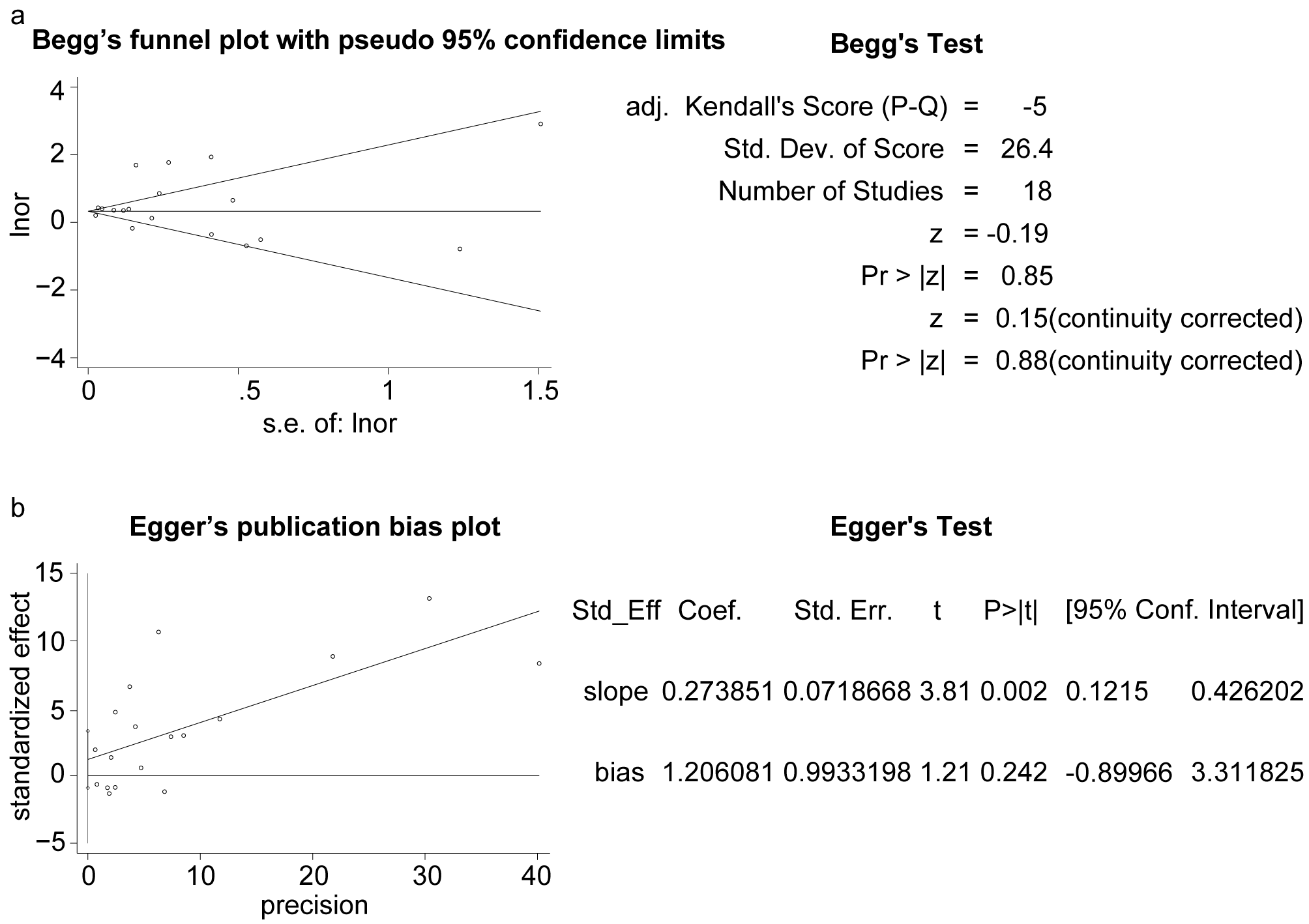


**a.** The horizontal line refers to pooled effect estimates. The two oblique lines indicate pseudo 95% confidence intervals. **b.** Egger’s test shows that there is no significant publication bias observed in the association between eczema and depression. The detailed information of these two tests was shown on the right side.
